# Effect of extraction method on the chemical profiles and bioactivities of soybean hull polysaccharides

**DOI:** 10.1002/fsn3.2483

**Published:** 2021-09-09

**Authors:** Lin Han, Hong Song, Licheng Fu, Jun Li, Lina Yang, He Liu

**Affiliations:** ^1^ College of Food Science and Technology Bohai University Jinzhou China

**Keywords:** antioxidant, extraction method, hepatoprotective, soybean hull polysaccharides

## Abstract

The yields, properties, and bioactivities of polysaccharides extracted by three methods from soybean hulls (SSCPs) were evaluated: hot water extraction (H‐SSCP), microwave‐assisted ammonium oxalate extraction (A‐SSCP), and microwave‐assisted sodium citrate extraction (S‐SSCP). A‐SSCP gave the highest yield of polysaccharides (9.3 ± 0.5%) although all three products had similar physicochemical characteristics and FT‐IR spectra. A‐SSCP and S‐SSCP produced polysaccharides with lower molecular weight distributions and higher total reducing power and scavenging ability for ABTS^+^• and DPPH• free radicals. Furthermore, the effect of SSCPs on carbon tetrachloride (CCl_4_)‐induced liver injury was investigated in the mice. When compared with H‐SSCP and S‐SSCP, A‐SSCP significantly decreased the levels of alanine aminotransferase (ALT), aspartate aminotransferase (AST), malondialdehyde (MDA), and reactive oxygen (ROS) to normal (*p* < .05) and increased the level of glutathione (GSH) to normal (*p* < .05). A‐SSCP was the most effective polysaccharide, yielding an approximately normal hepatic appearance with well‐preserved cytoplasm, obvious cell boundaries, with legible nuclei and nucleoli. This study indicates that polysaccharides extracted from soybean hulls via microwave‐assisted ammonium oxalate extraction have the potential to be developed as a new functional food contributing to the alleviation of liver damage.

## Graphical abstract

The polysaccharides extracted from soybean hulls via microwave‐assisted ammonium oxalate extraction have the potential to be developed as a new functional food contributing to the alleviation of liver damage.

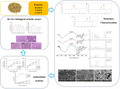

## 1 INTRODUCTION

Oxidation is a common reaction and an essential biological process in many organisms to produce energy. However, reactive free radical oxygen species can be produced in an uncontrolled manner during some in vivo oxidative reactions. These oxygen species can react with macromolecules, leading to diseases such as cancer, rheumatoid arthritis, and atherosclerosis (Finkel & Holbrook, 2000; Moskovitz et al., 2002). Synthetic antioxidants such as butylated hydroxyanisole and butylated hydroxytoluene are suspected of causing liver damage and being carcinogenic (Qi et al., 2005). It is necessary to develop naturally derived antioxidants to protect the human body against free radicals and retard the progress of various chronic diseases (Kinsella et al., 1994). Some natural plant polysaccharides with low cytotoxicity have been investigated as novel antioxidants in the food and pharmaceutical industries. Numerous studies have found that carbon tetrachloride (CCl_4_) exposure induces several alterations in liver tissues and that natural plant polysaccharides protect liver cells from such oxidative stress (Bargougui et al., 2019; Mahmoud et al., 2012).

Soybean, a species of legume, is one of the major sources of energy and nourishment for the world's population. Soy hulls are the seed coats of soybeans (~8% by weight) obtained as a by‐product of the soybean processing industry. Most are discarded or used in animal feeds (Kim et al., 2015); however, some studies have indicated that antioxidant components are present in plant hulls (Asamarai et al., 1996; Duh et al., 1992; Lai et al., 2010). The insoluble carbohydrate fraction of hulls consists of approximately 30% pectin, 20% cellulose, and 50% hemicellulos (Gnanasambandam & Proctor, 1999). Soy hulls have been considered as a source of novel polysaccharides. Soluble polysaccharides from soybean meal have been shown to have free radical scavenging activity and may be considered as good radioprotective agents (Mateos‐Aparicio et al., 2010; Yao et al., 2011). However, no information is available on the bioactivity of soy hull polysaccharides (SSCP). Our research group previously extracted SHSP with ammonium oxalate coupled with microwave treatment, concluding that SHSP consists mainly of galactose, xylose, galacturonic acid, arabinose, rhamnose, and glucose and that Mg^2+^ can induce the gelation of these polysaccharides (Liu et al., 2013). The aim of this study was to investigate the effects of various extraction methods on the yield of these polysaccharides. The chemical properties of SSCPs were characterized by high‐performance gel filtration chromatography (HPGFC), ion chromatography (IC), Fourier‐transform infrared spectroscopy (FT‐IR), and scanning electron microscopy (*SEM*). In addition, in vitro antioxidant activities and the in vivo hepatoprotective potential of SSCPs against CCl_4_‐induced tissue damage were evaluated. This information will be helpful in both exploiting a novel antioxidant and maximizing the utilization and value of soybean hulls.

## 2 MATERIALS AND METHODS

### 2.1 Materials and reagents

Soy hulls of Heihe 43 soybean were obtained from the Yu Wang Group in Shan Dong, China. ALT/AST Enzyme Activity Kit was purchased from Nanjing Jiancheng Bioengineering Institute. Ammonium oxalate, sodium citrate, vitamin C (Vc), monosaccharide standards, 1,1‐diphenyl‐2‐picrylhydrazyl (DPPH), 2, 2'‐azino‐bis (3‐ethylbenzothiazoline‐6‐sulfo‐nic acid) (ABTS), trifluoroacetic acid (TFA), phosphate buffer saline (PBS), and trichloroacetic acid (Metcalfe) were purchased from Sigma Chemical Co., Ltd. All other chemicals and reagents were analytical grade.

### 2.2 Extraction of SSCPs

#### 2.2.1 Hot water extraction

Fifty g of the soybean hull samples were broken into powder and discolored with ethanol solution (1%) in a ratio of 1:10 (m/v). The dried soybean bean hull samples were extracted with a ratio of soybean powder to water of 1:20 (m/v), 85℃ heating for 4 hr. After cooling and filtering, the extract was centrifuged at 4,500 × *g* for 10 min, the supernatant was collected and concentrated, and its pH was adjusted to 4.0 with 1 M HCl. The polysaccharide was precipitated by addition of 2 volumes of pure ethanol and dried at 65℃ for 6 hr, thus obtaining H‐SSCP samples.

#### 2.2.2 Microwave‐assisted ammonium oxalate/sodium citrate extraction

After discoloring with 1% ethanol solution, 50 g of the soy hull samples were extracted in 20 times volumes of 0.6% (w/v) ammonium oxalate/sodium citrate (analytical reagent) with 85℃ microwave heating for 20 min at 480 W. After cooling and filtering, the extracts were centrifuged at 4,500 × *g* for 10 min, the supernatant was collected and concentrated, and its pH was adjusted to 4.0 with 1 M HCl. Then, crude A‐SSCP/S‐SSCP were obtained by a series of procedure including ethanol precipitation and drying at 65℃.

### 2.3 Determination of carbohydrate, protein, sulfate, uronic acids, and acetyl group contents in SSCPs

The polysaccharide content can be quantified by phenol–sulfuric acid method (Cuesta et al., 2003). Coomassie Brilliant Blue G‐250 is used for investigating protein content (Georgiou et al., 2008). The sulfate content was determined by barium chloride–gelatin method (Kawai et al., 1969). Uronic acid units can be quantified by a colorimetric determination after treatment with concentrated sulfuric acid and carbazole (Li et al., 2007). The acetyl groups on the SSCPs are converted into a ferric‐acetohydroxamic complex that is quantitated using UV‐Vis spectrophotometer (Metcalfe, 2019).

### 2.4 Chemical characterization of SSCPs

#### 2.4.1 Molecular weight determination

The molecular weight of soybean hull polysaccharides was determined by using Waters 1525 HPGFC equipped with 2,414 differential refractive detector and Empower3 workstation. The soybean hull polysaccharide samples were dissolved in 0.1 mol/L NaNO_3_ ultrapure water solution, and its mass concentration was 10mg/ml. After filtration by microporous membrane, gel permeation filtration chromatography was used to analyze the sample, and the injection volume was 20 μL. Chromatographic analysis conditions: column: Ultrahydrogel™ Linear 300 mm ×7.8 mmid×2; flow rate: 0.9 ml/min; and column temperature: 45℃. The standard used for molecular weight correction curve is Dextran T series.

#### 2.4.2 Monosaccharide composition determination

5.0 mg of the soybean hull polysaccharide sample was dissolved in 4 mol/L TFA; it was hydrolyzed in oven at 110℃ for 6hr; methanol was added repeatedly and blow it dry with nitrogen blower; deionized water was used to fix volume to 25 ml; and the monosaccharide composition of soybean seed coat polysaccharide was analyzed by ion chromatography. The monosaccharide composition of soybean seed coat polysaccharide was analyzed by chromatograph. Determination conditions of ion chromatography: column: CarboPac PA20 column (150 mm ×3 mm); detector: pulse amperometric detector, gold electrode; injection volume: 25 μl; flow rate: 0.5 ml/min; and mobile phase: sodium hydroxide solution and sodium acetate solution.

### 2.5 FT‐IR and *SEM* analysis

A Tracer‐100 Fourier‐transform infrared spectrometer (Shimadzu, Japan) was used to evaluate the FT‐IR spectra of SSCPs. Briefly, 1 mg of the dry SSCP powder was mixed with 100 mg KBr powder and then pressed into tablet for FT‐IR measurement in the frequency range of 4000–400 cm−1.

The freeze‐dried SSCPs were fixed onto a copper stub. After sputtering with a layer of gold, the *SEM* images were observed and recorded using a S‐4800 *SEM* (JEOL Ltd., Japan) under a high vacuum condition at an accelerating voltage of 5 kV and image magnification of 30‐50k×.

### 2.6 Antioxidant activity assays

#### 2.6.1 DPPH radical scavenging activity

Various concentrations of SSCPs (2, 4, 6, 8, 10 mg/ml, previously dissolved in ethanol) were reacted with DPPH solution (0.004% w/v dehydrated ethanol) for 30 min at 25℃ in the dark followed by absorbance determination at 517 nm. Ascorbic acid was used as positive control. The DPPH radical scavenging rate was calculated by the following formula:

$$\text{Scavengingefficiency}\left(\%\right)=1‐\frac{A_{1}‐A_{2}}{A_{0}}\times 100\%$$

where A_0_: the absorbance without sample, A_1_: the absorbance of the sample and DPPH solution, and A_2_: the absorbance without DPPH.

#### 2.6.2 ABTS^+^ radical scavenging activity

Eighty‐eight μl (140 mmol/L) oxidant potassium persulfate solution was mixed with 5 ml (7 mmol/L) ABTS solution and stayed overnight under room temperature in dark conditions for ABTS raw liquor. ABTS solution was diluted with distilled water to 0.700 ~ 0.720 absorbance value at 734 nm, counting as A_0_.

0.1 ml polysaccharide solution in different concentrations (2, 4, 6, 8, 10 mg/ml) was admixed with 1.4 ml ABTS working fluid by a whirlpool oscillator and reacted in dark for 6 min at room temperature. The absorbance A_i_ at 734 nm was then determined. ABTS+radical scavenging rate is gauged in the light of the following formula:

$$\text{Scavengingefficiency}\left(\%\right)=\frac{1‐A_{i}}{A_{0}}\times 100\%$$

#### 2.6.3 Total reducing ability

In 25 ml plugged colorimetric tube, 2.5 ml of different concentrations (2–10 mg/ml) of polysaccharide solution, PBS buffer solution (0.2 mol/L, pH 6.6), and 1% potassium ferricyanide [K_3_Fe (CN)_6_] solution were added, respectively, and the mixture was retained in water bath at 50℃ for 20 min, cooled rapidly after reaction, and added 2.5 ml 10% trichloroacetic acid (Metcalfe). After the reaction, the supernatant was centrifuged at 4,000 r/min for 10 min. 2.5 ml of distilled water and 0.5 ml 0.1% of ferric chloride solution were then added into the test tube and let standing for 10 min. The absorption value A_i_ of mixing group at 700 nm wavelength was determined. Distilled water was used to determine the absorbance value A_0_. The larger the absorbance value, the stronger the reduction ability.

$$\text{Totalreductionability}=A_{i}‐A_{0}$$

### 2.7 Animal and experimental design

The Kunming mice (8 weeks old) were purchased from Jinzhou Medical University Laboratory Animal Center (Jinzhou, China). The mice were kept under controlled conditions of 12‐hr light/dark cycles at 22 ± 2℃ and 50%–55% relative humidity. All food, water, and experimental equipment used were sterilized. After a week acclimatization, mice were randomly divided into the following five groups (eight mice per group): normal control group (NC; receiving olive oil on day 8), model control group (MC; receiving CCl_4_ on day 8), A‐SSCP group (A‐Pro; receiving CCl_4_ on day 8 and 400 mg/kg body weight of A‐SSCP daily throughout), S‐SSCP group (S‐Pro; receiving CCl_4_ on day 8 and 400 mg/kg body weight of S‐SSCP daily throughout), and H‐SSCP group (H‐Pro; receiving CCl_4_ on day 8 and 400 mg/kg body weight of H‐SSCP daily throughout).

From days 1–7, mice in treatment groups NC group and MC group received sterilized distilled water by gavage administration once a day around 10 a.m.; mice in treatment groups A‐Pro, S‐Pro, and H‐Pro received 400 mg/kg bw body weight aqueous solution of A‐SSCP, S‐SSCP, and H‐SSCP, respectively, again once a day by intragastric administration at the above‐mentioned time. On the 8th day, mice in the NC group received a gavage injection of 1 ml/kg body weight olive oil; all other groups received an intragastric injection of 1 ml/kg body weight CCl_4_ (1:4, v/v, in olive oil). After 24 hr (day 9), NC group and MC group continued to receive the sterilized distilled water; A‐Pro, S‐Pro, and H‐Pro received polysaccharides for a further 7 days. The whole experiment was lasted for 16 consecutive days. At the end of the experiment, all mice were anesthetized with isoflurane gas, and under aseptic conditions, laparotomies were performed via a midline incision and livers harvested for enzyme activity evaluation. The blood samples were laid at 4℃ for 30 min and centrifuged at 2,000 × *g* and 4℃ for 20 min.

#### 2.7.1 Oxidative stress parameters in mice livers

The levels of alanine aminotransferase (ALT), aspartate aminotransferase (AST), malondialdehyde (Alemdar & Sain, 2008), glutathione (Huang et al., 2013), and reactive oxygen (ROS) were allied using the kits of Nanjing Jiancheng Bioengineering Institute. Each sample was assayed 3 times.

#### 2.7.2 Liver histopathology studies

Mice livers from all groups were removed and fixed immediately in 4% neutral buffered formalin, dehydrated in gradual ethanol (30%–100%), cleaned in xylene, and embedded in paraffin. Sections were prepared and stained with hematoxylin and eosin (H&E) for photo‐microscopic observation.

### 2.8 Statistical analysis

The data of all experiments were recorded as means ± standard deviations and were analyzed with SPSS (version 19.0, SPSS Inc.). Differences were considered significant at *p* < .05.

## 3 RESULTS AND DISCUSSION

### 3.1 Effects of extraction methods on yield of SSCPs

SSCPs were extracted by three methods, viz. hot water extraction (H‐SSCP), microwave‐assisted ammonium oxalate extraction (A‐SSCP), and microwave‐assisted sodium citrate extraction (S‐SSCP). The yields of SSCPs were as follows: H‐SSCP (1.2 ± 0.5%) < S‐SSCP (3.6 ± 0.4%) < A‐SSCP (9.3 ± 0.5%), indicating that microwave‐assisted ammonium oxalate/citrate extraction is a promising alternative technique for the production of SSCPs. Microwave irradiation of plant samples and short extraction times improve the yield of polysaccharides due to the heating of the solvent and rupturing of the cell walls which liberates polysaccharides from the matrix (Soria et al., 2015). Different extractants have significant effects on polysaccharide yield (Wang et al., 2019).

### 3.2 Effects of extraction methods on chemical composition of SSCPs

The main chemical components of SSCPs are summarized in Table 1. A‐SSCP and S‐SSCP contained low levels of sugar and protein, while H‐SSCP contained the highest, suggesting that hot water was preferable for the extraction of polysaccharide–protein complexes. A‐SSCP contained relatively high uronic acid, followed by S‐SSCP and H‐SSCP, suggesting that microwave‐assisted ammonium oxalate extraction was the best method for the preparation of neutral and acidic polysaccharides. These results demonstrate that sugar, protein, and uronic acid contents of SSCPs were significantly influenced by the extraction technique. The sulfate content of S‐SSCP and H‐SSCP was not significantly different, while A‐SSCP contained much higher levels. Within a certain concentration range, high sulfate content in polysaccharides may lead to higher antioxidant capacity (Chen & Huang, 2019).

**TABLE 1:** Chemical composition of SSCPs

SSCP	Protein (μg/mg)	Total sugar (μg/mg)	Uronic acid (μg/mg)	Sulfate (μg/mg)
A‐SSCP	34.7 ± 0.2^c^	216.5 ± 4.6^c^	730.4 ± 73.8^a^	328.6 ± 15.9^b^
S‐SSCP	46.0 ± 1.3^b^	296.2 ± 2.0^b^	534.9 ± 20.1^ab^	49.7 ± 0.6^a^
H‐SSCP	60.6 ± 0.6^a^	475.6 ± 6.1^a^	328.6 ± 15.9^b^	44.5 ± 1.5^a^

Note

Different letters above the bars indicate a significant difference in values at *p* < .05.

### 3.3 Molecular weight and monosaccharide composition of SSCP

Molecular weight reflects the molecular chain of polysaccharides and is closely related to biological activity. The molecular weight of the SSCPs is presented in Table 2 and Figure S1. The weight average molecular weight (Mw) and number average molecular weight (Mn) of H‐SSCP were significantly higher than for A‐SSCP and S‐SSCP. The Mw and Mn of A‐SSCP were 251 kDa and 41 kDa, respectively, which were higher than S‐SSCP (149 kDa and 36 kDa). The difference of molecular weight may be due to the different functional groups in the polysaccharide or the rearrangement or combination of polysaccharides in the extraction process. The molecular weights of these three SSCPs demonstrated considerable variation, suggesting that these differences caused by the extraction method might also affect their bioactivities (Liao et al., 2020). (Zeng et al., 2012) claimed that polysaccharides obtained from *Auricularia auricular* displayed significant antioxidant activity in a dose‐dependent pattern and to a greater extent than described by (Fan et al., 2007). The discrepancy in these reports may be due to differences in extraction method (microwave‐assisted versus. hot water extraction) and Mw of the extracted polysaccharides. The lower Mw of polysaccharides obtained by microwave‐assisted extraction probably led to increased antioxidant activity because of their higher number of free hydroxyl groups which can decrease viscosity and increase solubility of compounds (Mirzadeh et al., 2020).

**TABLE 2:** Characterization of SSCP composition

	A‐SSCP	S‐SSCP	H‐SSCP
Mw (kDa)	251	149	1,175
Mn (kDa)	41	36	463
Mw/Mn	6.1	4.1	2.5
Arabinose (%)	6.20	5.52	5.22
Rhamnose (%)	15.20	14.45	7.18
Galactose (%)	22.58	28.12	30.47
Glucose (%)	6.77	2.76	6.13
Xylose (%)	4.35	–	–
Mannose (%)	20.91	36.07	47.55
Galacturonic acid (%)	24.17	12.24	3.44

Plant polysaccharides are composed of different monosaccharides forming unique structures and properties. Mannose, galactose, arabinose, rhamnose, glucose, and galacturonic acid were the major monosaccharides in all three SSCPs, demonstrating notable homogeneity of monosaccharide types across the different extraction methods. However, the extraction methods significantly influenced monosaccharide concentrations (Table 2 and Figure S2). The molar ratios of mannose : galactose : rhamnose : arabinose : glucose : galacturonic acid for A‐SSCP were 3.1:3.3:2.5:1.1:1.0:3.3; for S‐SSCP 11.1:10.2:5.8:2.4:1.0:4.1; and for H‐SSCP 14.9:9.5:2.5:2.0:1.9:1.0. Mannose and galactose were the most abundant monosaccharides and constituted the backbone structure of the SSCPs. Many researches have indicated that monosaccharide ratio in the polysaccharides may lead to the observed differences in bioactivity. Several studies have indicated that galactose and mannose are highly relevant to bioactivity. Chen et al. also state that the low ratio of glucose in the glycosidic structure may enhance antioxidant capacity (Chen et al., 2010). The monosaccharide contents of probiotic exopolysaccharides influence their anti‐inflammatory activity toward macrophages, with high galactose >high rhamnose >high glucosamine (Chen et al., 2019). Other reports show that the rhamnose content can enhance the antioxidant ability through radical scavenging (Zhang et al., 2016); moreover, the polysaccharides from Ganoderma lucidum have a higher ability to regulate immunoglobulin G levels with a higher proportion of glucose (Ji et al., 2020). The more types of monosaccharides present, the higher the antioxidant and the immunostimulating activities of Ganoderma lucidum polysaccharides (Shi et al., 2013).

### 3.4 Fourier‐transform infrared spectroscopic analysis

**FIGURE 1:**
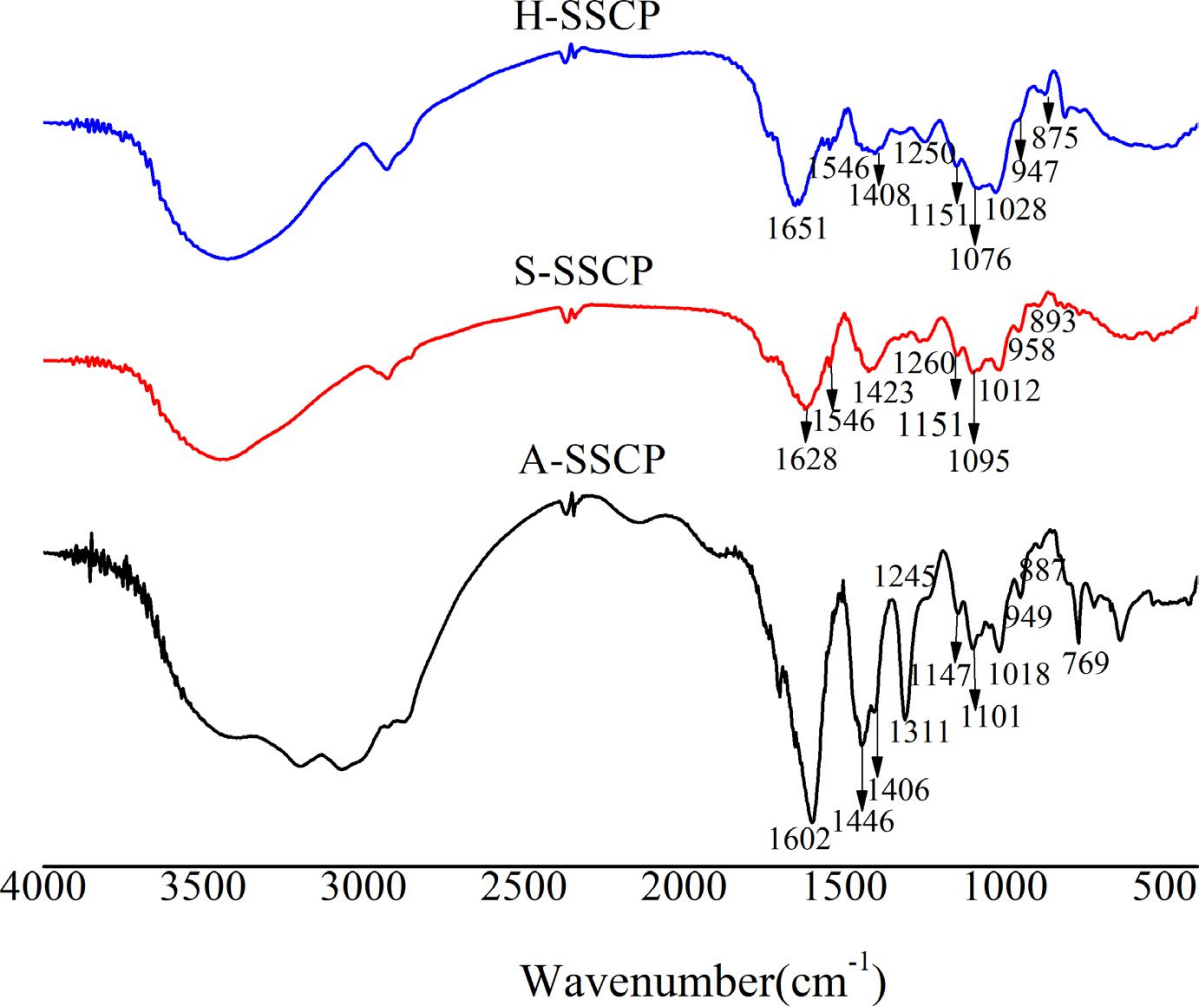
FT‐IR spectra of polysaccharides A‐SSCP, S‐SSCP, and H‐SSCP

FT‐IR spectra of A‐SSCP, S‐SSCP, and H‐SSCP are illustrated in Figure 1. The main absorption bands were similar in all three polysaccharides. The peaks around 3,300 and 2,970 cm^−1^ are due to the stretching vibration of a hydroxyl group and a C‐H bond, respectively, indicating that the SSCPs possess these groups which are typical of polysaccharides. The absorption peaks observed at 1602–1651 cm^−1^ and 1400–1450 cm^−1^ are assigned, respectively, to the asymmetric and symmetric stretching of carboxylate anion groups (Yan et al., 2018). The appearance of amide group vibrations at 1,408 cm^−1^ (due to Amide III), 1546 cm^−1^ (due to Amide II), and 1651 cm^−1^ (due to Amide I) indicates that bound protein is present in S‐SSCP and H‐SSCP, which is consistent with the results of Gu et al. (Hao et al., 2020). There are also absorption peaks at 1,250 cm^−1^ arising from asymmetrical S = O stretching vibrations of sulfate groups in S‐SSCP and H‐SSCP. In addition, the peaks between 1,000 and 1,200 cm^−1^ (specifically 1,095 and 1,151 cm^−1^) are consistent with ring vibrations overlapping with stretching vibrations of C‐O‐H side groups and C‐O‐C glycosidic bond vibrations, suggesting that the three polysaccharides contain pyranose monomers in their structures. Moreover, the characteristic band at 896 cm^−1^ indicates a β‐anomeric configuration and the characteristic bands at 956 and 873 cm^−1^ are attributable to a pyranoid ring.

### 3.5 *SEM* of SSCPs

**FIGURE 2:**
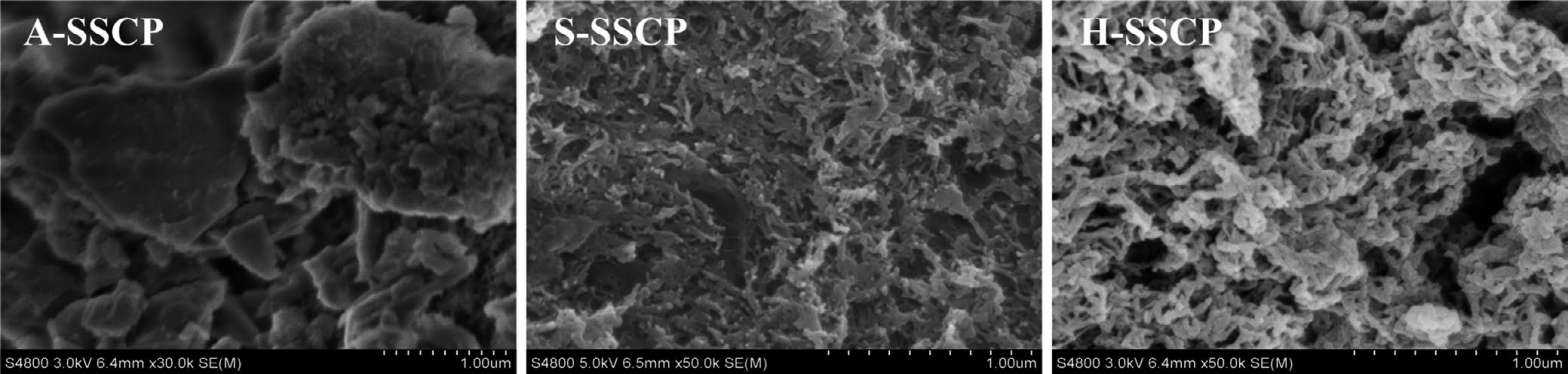
*SEM* micrographs of SSCPs obtained by different extraction methods

*SEM* is a useful tool for observing surface features in the micron to submicron range on polymeric materials. As shown in Figure 2, A‐SSCP morphology was significantly different from the other polysaccharides. The surface of A‐SSCP was smooth with a large sheet structure. In contrast, S‐SSCP and H‐SSCP were dispersed into relatively loose, irregular fibrous strips. This difference can be attributed to the differing monosaccharide compositions, particularly uronic acid, the protein content, and different glycosidic links within the three SSCPs (He et al., 2018; Ji et al., 2020).

### 3.6 In vitro antioxidant activity assays

#### 3.6.1 DPPH radical scavenging activity assay

**FIGURE 3:**
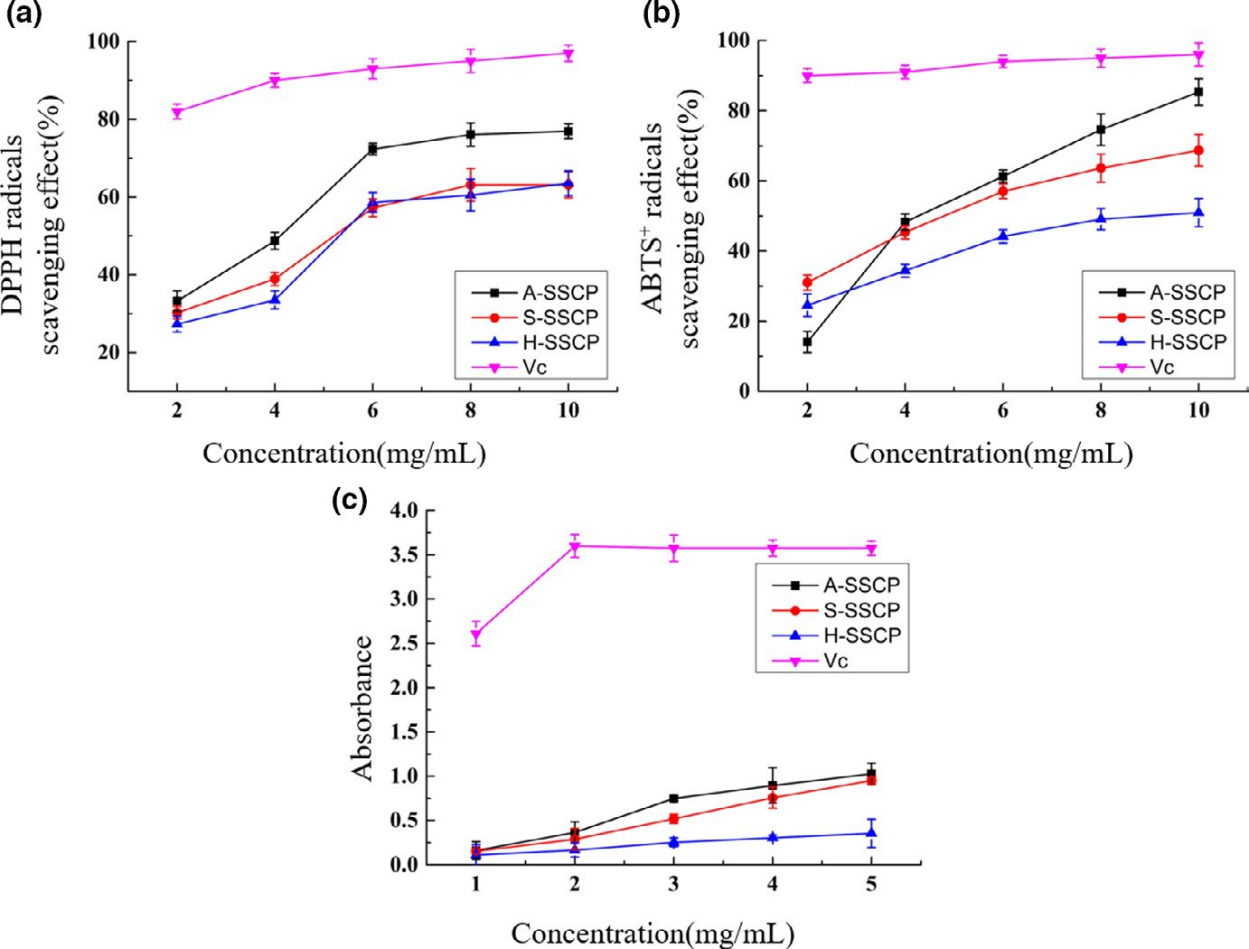
In vitro antioxidant activity of SSCPs: (a) DPPH radical scavenging activity, (b) ABTS^+^ radical scavenging activity, and (c) total reducing ability. Data are mean ± standard error of the mean (*n* = 3)

DPPH is a stable free radical molecule centered on a nitrogen atom with an unpaired electron. It absorbs strongly at 517 nm. The abundance of this free radical readily decreases following exposure to proton radical scavengers. A deep‐purple solution of DPPH changes to yellow as it is quenched by antioxidants. DPPH is the most common free radical used to determine antiradical and antioxidant activities of natural polysaccharides. As shown in Figure 3a, all the SSCPs displayed a significant dose‐dependent increase in radical scavenging activity at concentrations of 2–10 mg/ml. The DPPH radical scavenging effects of A‐SSCP, S‐SSCP, and H‐SSCP at 10 mg/ml were 76.9 ± 1.9%, 63.6 ± 3.3%, and 63.1 ± 3.2%, respectively, and all were lower than Vc (97.0 ± 2.1%).

#### 3.6.2 ABTS^+^ radical scavenging activity

ABTS^+^ radicals are widely used for the determination of total antioxidant capacity. The ABTS^+^ radical scavenging capacity of SSCPs from different extraction methods is shown in Figure 3b. All three SSCPs exhibited significant concentration‐dependent ABTS^+^ scavenging ability. As SSCP concentration increased, the scavenging effect of A‐SSCP increased from 14.0 ± 3.0% to 85.3 ± 3.8%. S‐SSCP and H‐SSCP at 10.0 mg/ml reached 68.7 ± 4.5% and 50.9 ± 4.0%, respectively, less than Vc (96.0 ± 3.3%), but similar to results reported by Zhang et al. In addition, SSCPs were more effective at scavenging ABTS^+^ radicals than DPPH radicals. This may be because the DPPH scavenging assay is more applicable for evaluating hydrophobic antioxidants, while the ABTS^+^ scavenging assay is more suitable for evaluating hydrophilic antioxidants (Liao et al., 2015).

#### 3.6.3 Total reducing ability

The reducing ability of a compound can serve as a significant indicator of its potential antioxidant activity. Figure 3c illustrates the reducing power of A‐SSCP, S‐SSCP, and H‐SSCP. Higher absorbance is generally associated with greater reducing power. In the concentration range 1–5 mg/ml, all the SSCPs displayed significant reducing power. A‐SSCP and S‐SSCP exhibited similar reducing power, both being higher than H‐SSCP at all concentrations tested. At 5.0 mg/ml, the reducing capacities of A‐SSCP, S‐SSCP, and H‐SSCP, as indicated by absorbance, were 1.03 ± 0.12, 0.95 ± 0.04, and 0.35 ± 0.16, respectively.

The SSCPs possessed different DPPH and ABTS^+^ radical scavenging activities and total reducing abilities. This may be due to the free radical system used for antioxidant evaluation. Different systems have major differences in the mechanisms of their determination, and the polysaccharides may have specific scavenging effects due to an affinity between the antioxidant and the substrate used in the assay (Song et al., 2019). A‐SSCP, with its high antioxidant capacity, may have a higher density of hydroxyl groups which will increase its reductive capacity compared with H‐SSCP which has a higher content of acetyl groups acting as the electron withdrawing moiety (Korcova et al., 2015). The presence of electrophilic groups such as ketones and aldehydes in acidic polysaccharides can improve radical scavenging ability through facilitating the release of hydrogen from O‐H bonds. Hence, the enhanced hydrogen‐donating ability of high concentration polysaccharides is related to the high number of unmethylated uronic acid and hydroxyl groups. Maintaining the integrity of the triple helical structure of extracted polysaccharides with lower Mw can also significantly increase their potential for scavenging free radicals. Under such conditions, smaller polysaccharide fractions have a greater chance to come in contact with free radicals due to their larger surface area (Mirzadeh et al., 2020). The presence of –OSO_3_H groups in the polysaccharide could also activate the hydrogen atom of an anomeric carbon, giving it a stronger donating capacity (Song et al., 2018). This may be one of the most important reasons that A‐SSCP exhibited greater antioxidant activity than S‐SSCP and H‐SSCP.

### 3.7 Correlation analysis

The relationships between the composition of the three polysaccharides and their antioxidant capacity were elucidated by correlation analysis as shown in Table 3. DPPH radical scavenging activity was significantly (*p* < .01) and positively correlated with arabinose and galacturonic acid content and negatively correlated with galactose and mannose content. It can be concluded that the antioxidant capacity of these polysaccharides may be closely related to their unique structure and monosaccharide contents. ABTS^+^ scavenging data were very similar to DPPH except for rhamnose content which had a weaker influence on DPPH radical scavenging activity. The contents of rhamnose and galacturonic acid were positively related to total antioxidant capacity (as indicated by total reducing ability), the increase in rhamnose and galacturonic acid contents enhancing total reducing ability. The differing physicochemical properties of the three polysaccharides produced different free radical scavenging profiles.

**TABLE 3:** Correlation analysis (R values) of polysaccharide composition and antioxidant capacity

	DPPH radicals	ABTS^+^ radicals	Total reducing ability
DPPH radicals	1		
ABTS^+^ radicals	0.9^a^	1	
Total reducing ability	0.7^b^	0.9^a^	1
Mannose	−0.8^a^	−0.9^a^	−0.8^a^
Galactose	−0.9^a^	−0.9^a^	−0.7^b^
Rhamnose	0.6	0.8^a^	0.9^a^
Arabinose	0.9^a^	0.9^a^	0.7^b^
Glucose	0.4	0.06	−0.2
Galacturonic acid	0.8^a^	0.9^a^	0.8^a^
Mw	−0.5	−0.8^a^	−0.8^a^
Protein	0.8^a^	0.9^a^	0.9^a^
Sulfate	0.9^a^	0.8^a^	0.6

^a^ Correlation is significant at *p* < .01 level.

^b^ Correlation is significant at *p* < .05 level.

Molecular weight was significantly (*p* < .01) and negatively correlated with ABTS^+^ scavenging activity and total reducing ability. It can be concluded that the antioxidant capacity of polysaccharides may be closely related to, and weakens with, an increase in molecular weight. Protein content was significantly (*p* < .01) and positively correlated with DPPH scavenging, ABTS^+^ scavenging, and total antioxidant capacity. In addition, the sulfate content of the polysaccharides correlated positively with their DPPH and ABTS^+^ radical scavenging activity.

In summary, the higher antioxidant capacity of A‐SSCP compared with S‐SSCP and H‐SSCP may be attributed to the higher contents of arabinose, galacturonic acid, sulfate, the lower protein content, and low Mw. This is in accordance with the conclusions of (Mirzadeh et al., 2020).

### 3.8 In vivo biological activity assays

#### 3.8.1 Effect of SSCPs on ALT and AST activity

**FIGURE 4:**
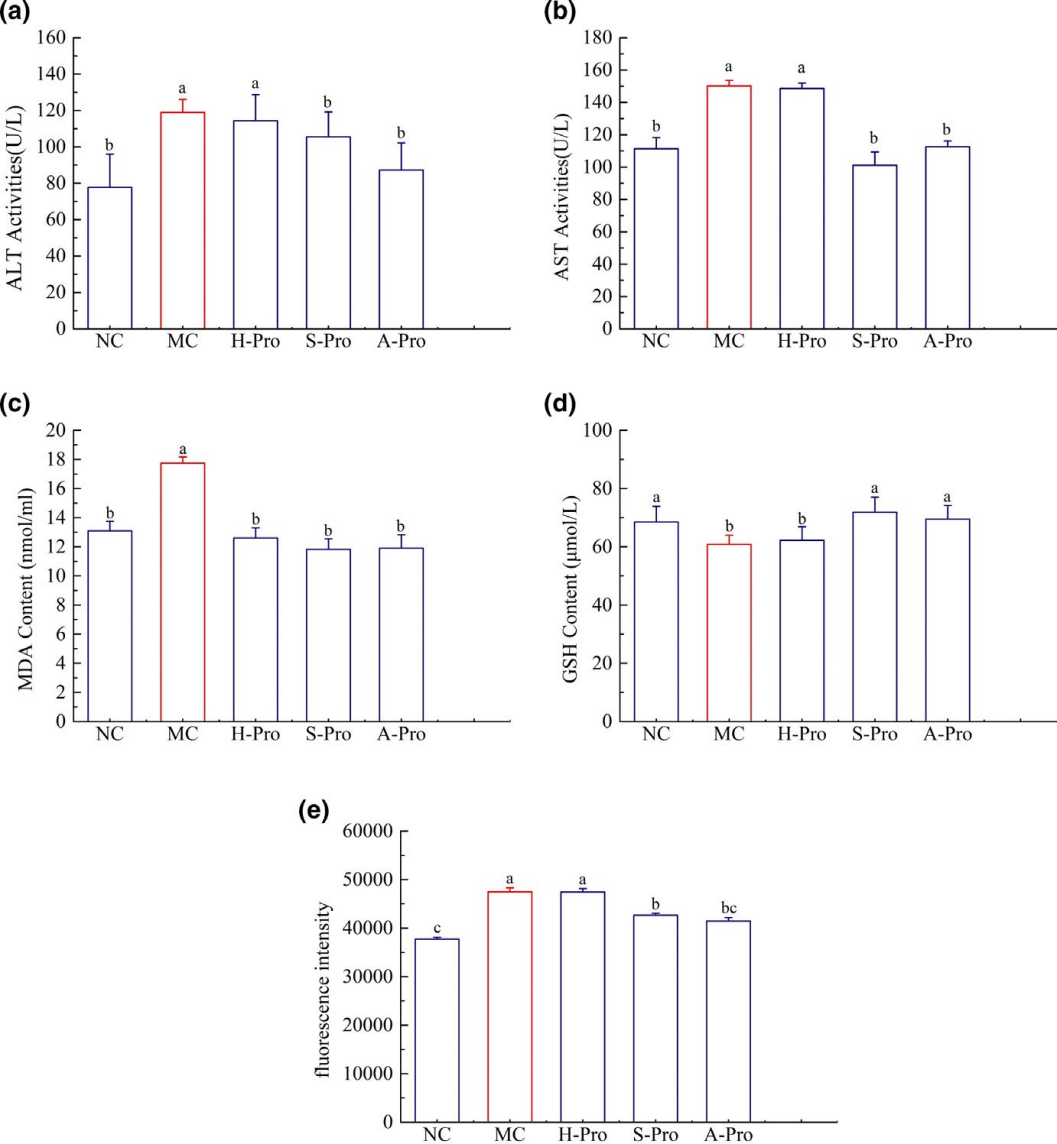
Effect of SSCPs on (a) ALT activity, (b) AST activity, (c) MDA content, (d) GSH content, and (e) fluorescence intensity of ROS in mouse serum. Data are mean ± standard error of the mean (*n* = 8). Columns with different letters differ significantly (*p* < .05)

Hepatoprotective activity of SSCPs against CCl_4_‐induced liver damage in mice was also evaluated by analyzing serum ALT and AST activity (Figure 4). Compared to the NC group, significant elevation of ALT and AST enzyme activities was seen in the MC group, indicating successful construction of a hepatotoxicity mouse model. Serum activities of ALT and AST were significantly decreased in mice treated with A‐SSCP and S‐SSCP compared with the MC group, while H‐SSCP treatment had no significant effect on their activities. This indication that A‐SSCP and S‐SSCP have potential to protect against CCl_4_‐induced liver damage is in accordance with the report of Hamid et al. (Hamid et al., 2017).

#### 3.8.2 Effects of SSCPs on GSH, MDA and ROS

We investigated the impact of SSCPs on oxidant stress status in CCl_4_‐injected liver by measuring the content of (GSH, MDA and ROS). Obviously, injection of CCl_4_ could lead to lower GSH content (*p* < .05), and elevate MDA contents (*p* < .05) when compared to that in the NC group (Figure 4c–e). However, this abnormal change can be mitigated by treatment with A‐SSCP and S‐SSCP. Moreover, the ROS contents showed a significant decrease in the administration of A‐SSCP/S‐SSCP at 400 mg/kg body weight. These results indicated that A‐SSCP/S‐SSCP treatment was able to inhibit lipid peroxidation and inhibit the increasing of ROS and stimulate the production of nonenzyme antioxidant glutathione in CCl_4_‐injured liver.

#### 3.8.3 Histopathological examination

**FIGURE 5:**
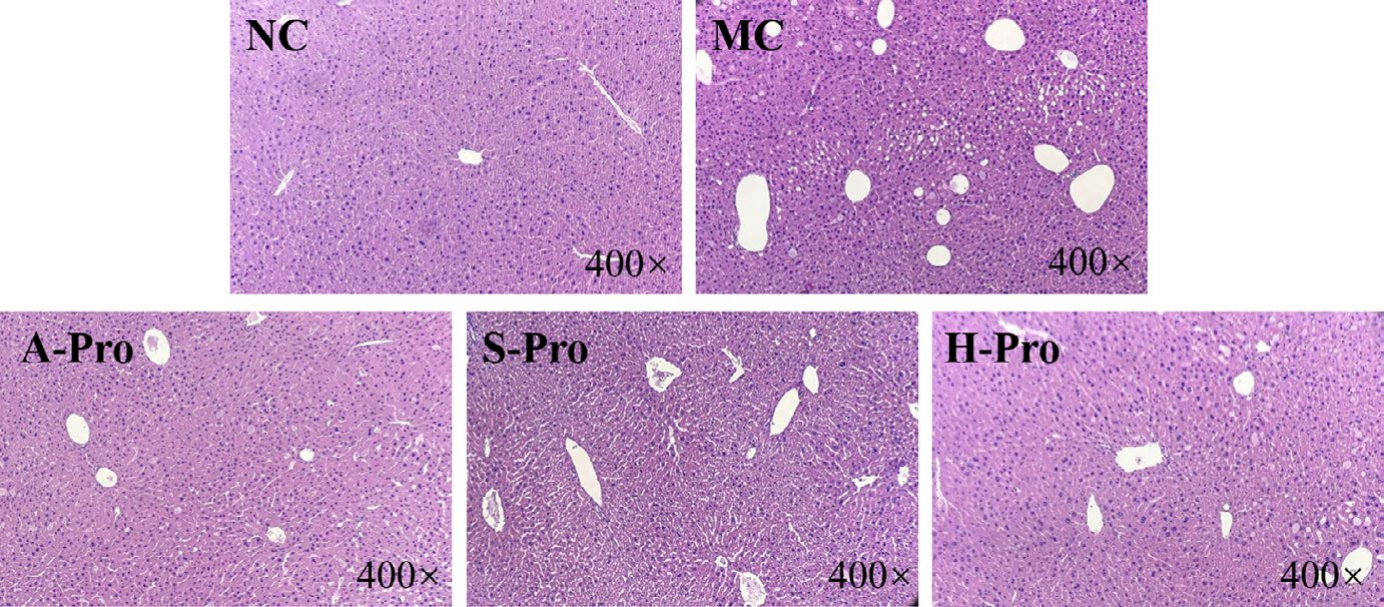
Histopathological effects of SSCPs on mice liver hepatocytes stained with H&E (400×)

The histopathological appearance of the livers of control and treated mice is shown in Figure 5. Liver sections from the NC group showed normal hepatocytes with preserved cytoplasm and nucleus. In contrast, the MC group revealed large, dense fat vacuoles in the tissue and liver damage indicating moderate to severe hepatocellular degeneration. Compared with the MC group, the number and scale of fat vacuoles in the polysaccharide treatment groups (A‐Pro, S‐Pro, and H‐Pro) were reduced. A‐SSCP was the most effective polysaccharide, yielding an approximately normal hepatic appearance with well‐preserved cytoplasm, obvious cell boundaries, with legible nuclei and nucleoli (Barros et al., 2018; Chidambaram & Carani Venkatraman, 2010; Tian et al., 2012). These histopathological results, combined with the antioxidant assays and biochemical tests, suggest that A‐SSCP can protect liver tissue from CCl_4_‐induced hepatic damage.

## 4 CONCLUSION

The influence of extraction methods of SSCPs from soybean hulls was studied. A‐SSCP had the highest yield compared with S‐SSCP and H‐SSCP but all displayed typical polysaccharide characteristics. All three exhibited similar monosaccharide profiles but significantly different concentrations. A‐SSCP showed higher antioxidant activity than S‐SSCP and H‐SSCP, which was attributed to its higher content of arabinose, galacturonic acid, sulfate, lower protein content, and low Mw. A‐SSCP also exhibited considerable protective effects against CCl_4_‐induced liver damage in mice. This study indicates that polysaccharides extracted from soybean hulls via microwave‐assisted ammonium oxalate extraction have the potential to be developed as a new functional food contributing to the alleviation of liver damage (Chen & Huang, 2019; Guo et al., 2019; Yue et al., 2014; Zhang et al., 2018).

## CONFLICT OF INTEREST

The authors of this manuscript state that they do not have conflict of interest to declare.

## AUTHOR CONTRIBUTIONS

**Lin Han:** Data curation (equal); Investigation (equal); Methodology (equal); Software (equal); Validation (equal); Writing‐original draft (equal). **Hong Song:** Conceptualization (equal); Data curation (equal); Formal analysis (equal); Methodology (equal); Project administration (equal); Software (equal); Supervision (equal); Validation (equal); Writing‐original draft (equal); Writing‐review & editing (equal). **Licheng Fu:** Data curation (equal). **Jun Li:** Formal analysis (equal); Software (equal). **Linan Yang:** Conceptualization (equal); Data curation (equal); Software (equal). **He Liu:** Funding acquisition (equal); Project administration (equal); Resources (equal); Writing‐review & editing (equal).

## ETHICAL APPROVAL

All animals were housed and cared for in accordance with the Chinese Pharmacological Society Guidelines for Animal Use.

## Supporting information

Fig S1‐S2
