# Supplementary material for: Effect of extraction method on the chemical profiles and bioactivities of soybean hull polysaccharides
Source: Food Sci Nutr. 2021 Sep 9;9(11):5928–38. doi: 10.1002/fsn3.2483 (PMC8565245; doi:10.1002/fsn3.2483)
Supplement: Supplementary file 1 — Fig S1‐S2 [file FSN3-9-5928-s001.docx]

Supplementary materials

Effect of extraction method on the chemical profiles and bioactivities of soybean hull polysaccharides

Lin Han ^1, a^, Hong Song ^1, a,*^, Licheng Fu ^1^, Jun Li ^1^, Lina Yang ^1^, He Liu ^1, *^

^1^ College of Food Science and Technology, Bohai University, Jinzhou, 121013, China

^*^ Correspondence

Hong Song, College of Food Science and Technology, Bohai University, Jinzhou, 121013, China.

E-mail address: [songhong8912@163.com](mailto:songhong8912@163.com)

He Liu, College of Food Science and Technology, Bohai University, Jinzhou, 121013, China.

E-mail address: [liuhe2066@163.com](mailto:liuhe2066@163.com)

^a^ Lin Han and Hong Song contributed equally to this work


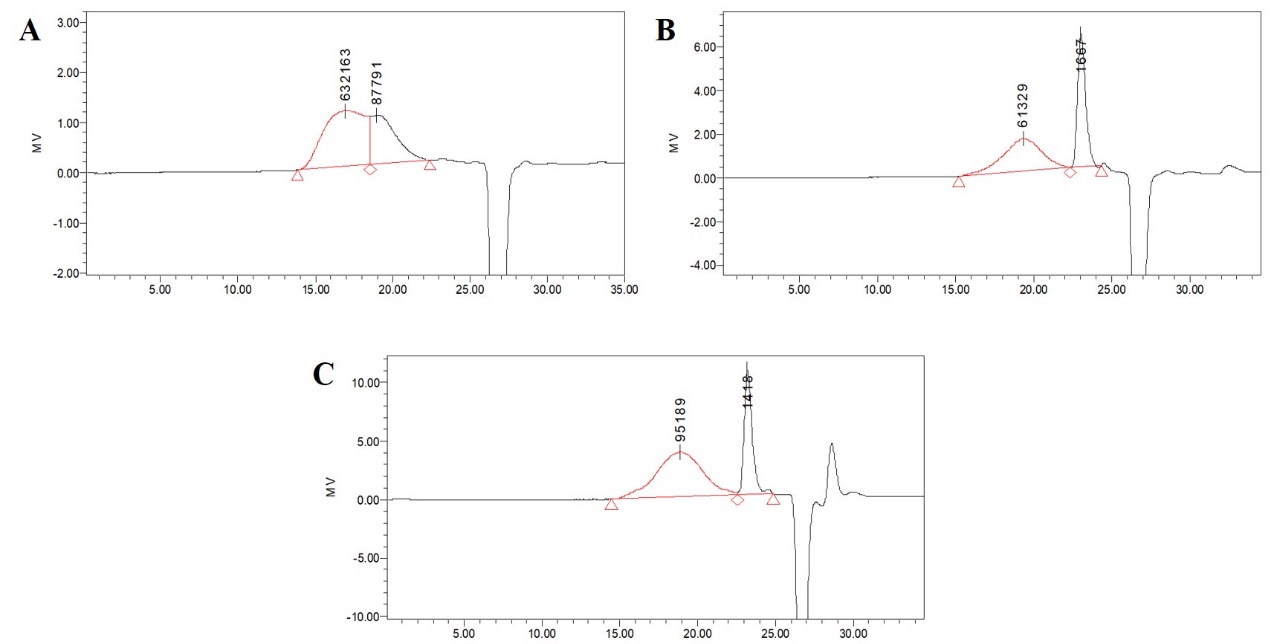


**FIGURE S1** Molecular weight of the SSCPs. (A) A-SSCP, (B) S-SSCP, (C) H-SSCP


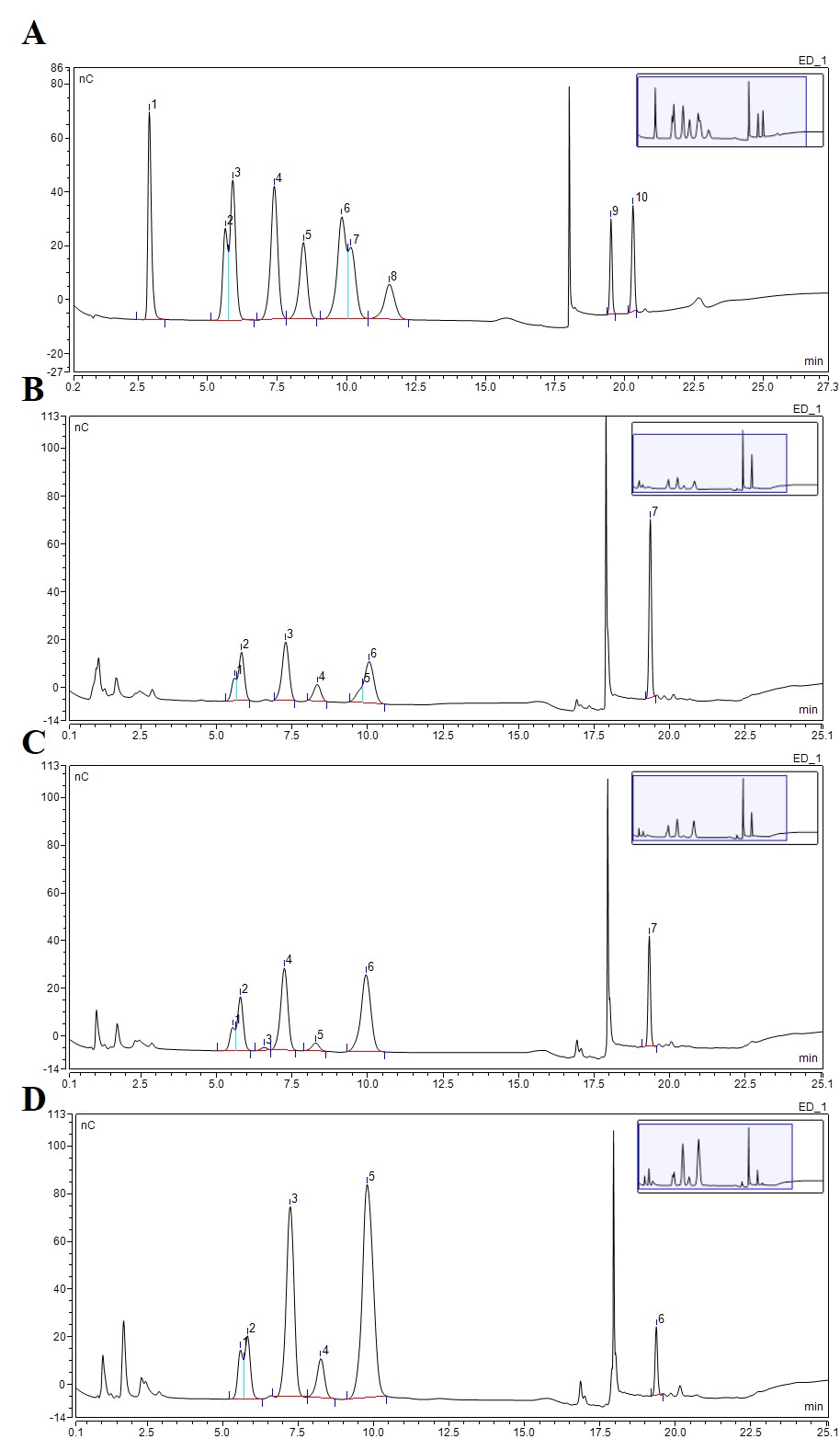


**FIGURE S2** Monosaccharide content of SSCPs, (A) monosaccharide standard (1 : fucose, 2 : arabinose, 3 : rhamnose, 4 : galactose, 5 : glucose, 6 : xylose,7 : mannose, 8 : fructose, 9 : galacturonic acid, 10 : Glucuronic acid),（B）A-SSCP, (C) S-SSCP, (D) H-SSCP
